# Belief in optimism might be more problematic than actual optimism

**DOI:** 10.3389/fpsyg.2014.00624

**Published:** 2014-06-19

**Authors:** Michael M. Roy

**Affiliations:** ^1^Department of Psychology, Elizabethtown College, Elizabethtown, PA, USA; ^2^Department of Music, North-West UniversityPotchefstroom, South Africa

**Keywords:** time, optimism, bias, prototype, memory, future

People appear to be biased in their predictions of the future; predicting that projects will take less time than they actually will (see Roy et al., 2005a; Buehler et al., 2010; Halkjelsvik and Jørgensen, 2012, for review) and that they will be unlikely to experience future misfortunes (see Dunning et al., 2004, for review). These biases are frequently attributed to people being overly optimistic (Armor and Taylor, 1998, 2002; Dunning et al., 2004). However, research indicates that many of these seemingly motivated biases could actually be due to task characteristics or other non-motivational causes (Juslin et al., 2000; Chambers and Windschitl, 2004; Roy et al., 2005a). Here I review research, focusing on my own work on time estimation and self-assessment, which indicates that these seemingly optimistic biases might often have other non-motivational causes. Further, I discuss why a belief that people are overly optimistic might be problematic.

## Optimism in time estimation?

The tendency for people to think that they will finish tasks earlier than they actually will is frequently thought of as fitting into a larger category of optimistic biases (Armor and Taylor, 1998, 2002). It has been proposed that underestimation is due to people forming an optimistic scenario of how a task will be completed and ignoring memory for how long similar tasks have taken in the past (Kahneman and Tversky, 1982; Buehler et al., 2002).

However, underestimation of future task duration might not be due to optimism about the future, but instead be due to biased memories of the past (Roy et al., 2005a; see also Griffin and Buehler, 2005; Roy et al., 2005). Similarly, it has been proposed that it is the quality of people's episodic (Szpunar et al., 2013) and semantic (Irish and Piguet, 2013) memories that predict their ability to envision the future (Schacter et al., 2008; Szpunar and McDermott, 2008). In support of the view that biased predictions are caused by biased memories, research indicates that factors that influence memories of past task duration have parallel influences on predictions of future task duration (Roy and Christenfeld, 2007, 2008) with biased memories frequently associated with biased predictions (Thomas et al., 2004, 2007). Furthermore, correcting memory for past task duration is one of the only interventions that has been found to successfully reduce bias and improve accuracy for predictions of future task duration (Roy et al., 2008). In relation, people seem to be accurate in estimates for tasks where frequent feedback on timing is given (Tobin and Grondin, 2012), but not for tasks where feedback is absent (Tobin and Grondin, 2009; Tobin et al., 2010; Bisson et al., 2012). In the field of software development, predictions of for how long a new project will take are more accurate when data on task duration for similar projects is utilized during predictions than when it is not (Jørgensen, 2004a; Furulund and Moløkken-Østvold, 2007).

While bias in predicted duration appears to often be due to bias in memory, memory itself could be biased by people's motivations. However, bias in remembered duration often appears to be due to task characteristics such as novelty (Boltz et al., 1998; Hinds, 1999; Roy and Christenfeld, 2007; Tobin et al., 2010), relative duration (Yarmey, 2000; Roy and Christenfeld, 2008; Tobin and Grondin, 2009), size of potential estimation anchors (Thomas and Handley, 2008), and duration since completion (Roy et al., 2008). These tasks characteristics may alter attention that is paid to the task (Thomas and Weaver, 1975) or memory storage size associated with the task (Ornstein, 1969; Block and Reed, 1978) and bias estimation. Task characteristics cause bias in remembered duration that in turn causes bias in predicted duration. For example, actors and observers appeared to be more influenced in their estimates, for both past and future tasks, by task characteristics, such as overall task duration and number of remembered task components, than they were by being the more motivationally involved person performing the task (Roy et al., 2013a; see also Byram, 1997; Hinds, 1999; Jørgensen, 2004b).

The reason that memory and prediction might be similarly biased is that both might rely on a similar constructive process with estimation based upon a general prototypical representation for task duration that is adjusted up or down depending on the specifics of the situation (Burt and Kemp, 1994; Roy et al., 2005a). Because memory for past task duration is often biased (see Wallace and Rabin, 1960; Fraisse, 1963; Block and Zakay, 1997 for reviews), the prototypical representations that people have for many tasks, which is the average of previous experience, is similarly biased. A result of prototypical memories that underestimate duration is an expectation that tasks in the future, such as travel plans, will take less time than they will in actuality (van de Ven et al., 2011). While people frequently underestimate how long tasks will take in the future, much of this bias appears to be due to task characteristics and not due to optimism or motivation. This is not to say that motivation never plays a role in bias; at times people's motivations influence their memories for past task duration (Meade, 1963; Schwab et al., 2013) and their predictions of future duration (Buehler et al., 1997; Byram, 1997). However, research indicates that bias in estimated duration frequently exists without motivational causes.

## Optimism in other judgments?

A person's estimate of task duration is often tied to their perceived competency at the task. For many tasks, quicker completion is linked to high ability. Indeed, more experience with a task is often linked to a greater tendency to underestimate task duration (Boltz et al., 1998; Hinds, 1999; Roy and Christenfeld, 2007). Further, people often appear to be overly optimistic in their self-assessments, rating themselves as above average on a number of skills and personality traits (see Taylor and Brown, 1988; Chambers and Windschitl, 2004; Dunning et al., 2004; Sedikides and Gregg, 2008, for reviews). It would seem logically impossible for the majority of people to be above average (Taylor and Brown, 1988). However, similar to time estimation, this apparent bias may be due to people relying on prototypical representations of ability when assessing their own abilities (Krueger, 1998; Gigerenzer, 2002; Moore, 2007; Galesic et al., 2012; Roy et al., 2013b). People understand that various skills have skewed ability distributions and their self-assessments are often related to distribution shape: high when most are believed to be good and low when most are believed to be bad (Galesic et al., 2012; Roy et al., 2013b). When the skill being rated has a skewed distribution, a tendency to rely on prototypical representations can lead to self-assessments that, on face value, appear to indicate a belief in the self as being falsely unique (above or below average), but actually may indicate a belief in the self as being prototypical (near the mode of the distribution; Roy et al., 2013b). Further, a reliance on prototypes can also help explain why people underestimate the likelihood that they will experience rare events, such as getting cancer, and overestimate the likelihood that they will experience common events, such as owning a car (Chambers et al., 2003; Kruger and Burrus, 2004).

It is also possible that other task characteristics, such as how clearly defined and specific the task is (Dunning et al., 2004; Roy and Liersch, 2013) or how much information people have about their own ability and the ability of others (Chambers and Windschitl, 2004), can influence self-assessments. On a related note, people's overconfidence in their responses on quizzes and tests might have more to do with the type of questions being asked than with actual self-confidence (see Juslin et al., 2000 for review). While people appear to overestimate their abilities and performance, these biases appear to often be due to specific aspects of the skill, performance or event and not due to optimism or motivation.

## Problems with perceived optimism

While people might not be overly optimistic about how their future will unfold, a *belief* in optimism as the cause of bias may be problematic both for researchers and for people in general. Inasmuch as biased predictions are viewed as being caused by optimism and that being optimistic is viewed positively, people might not seek out the true causes of bias and ways to eliminate bias.

Even though people acknowledge they are often biased in their predictions (Buehler et al., 2002; Armor et al., 2008), it is not clear if they know why they are biased. In general, people are not always able to accurately describe their decision-making processes (e.g., Wilson and Hodges, 1992). It appears that, like researchers (Taylor and Brown, 1988; Armor and Taylor, 1998, 2002; Dunning et al., 2004), people often attribute their errors to being overly optimistic (Armor et al., 2008). The belief in optimism as a cause of bias can be problematic for two reasons: first, people might be missing the real causes of their error. To make accurate and unbiased predictions for how long it will take to complete a task, people would need to realize that factors such as task length (Roy and Christenfeld, 2008), familiarity (Roy and Christenfeld, 2007), and complexity (Roy et al., 2013a) biased their memories for these tasks and correct these memories before estimating future task duration. Simply ascribing their error to optimism would make them miss the actual causes.

Second, while optimism appears to be a popular (Christensen-Szalanski and Beach, 1984), convenient, and more importantly, common excuse for error, there are few negatives to being seen as overly optimistic. While people believe that they are at times overly optimistic, they actually believe that they should try to be more optimistic (Armor et al., 2008). They appear to be willing to deal with what they believe are the negative consequences of their optimism. To a certain degree, they would be correct to do so because a large number of positive outcomes have been associated with having an optimistic outlook (e.g., Rasmussen et al., 2009). Because they do not mind their self-diagnosed cause of their bias, people will not be motivated to seek out the real cause. Interventions aimed at decreasing bias by decreasing optimism are likely to be unpopular as well as misguided (Roy et al., 2005a).

## Summary

People often appear to be overly optimistic in their predictions of the future. However, much of this apparent optimism could be due to other processes such as an over reliance on prototypical representations. In the case of time estimation, people's representation for task duration is often too short, causing them to underestimate future completion times. To improve predictions, factors that bias memory need to be taken into account and corrected. If, instead, bias in prediction is attributed to an optimistic outlook, then people will be unlikely to identify and correct the true causes of their bias. Interventions might be better aimed at decreasing the *belief* in over-optimism, not optimism itself.

### Conflict of interest statement

The author declares that the research was conducted in the absence of any commercial or financial relationships that could be construed as a potential conflict of interest.
